# Factors Associated with Invasive Coronary Angiography Use in Type 2 Myocardial Infarction: A Systematic Review with Narrative Synthesis

**DOI:** 10.3390/jcm15197586

**Published:** 2026-09-30

**Authors:** Ngoc Thai Kieu, Mays Tawayha, Manoj Sharma, Aliza Yaqub, Noman Chaudhary, Ahmed Ali, Husam Katib, Wassim Mosleh

**Affiliations:** 1 Department of Internal Medicine, Mercy Hospital of Buffalo, University at Buffalo, Buffalo, NY 14220, USA; 2 Department of Geriatric Medicine, HCA Florida Aventura Hospital and Nova Southeastern University, Aventura, FL 33180, USA; 3 Department of Interventional Cardiology, Mercy Hospital of Buffalo, University at Buffalo, Buffalo, NY 14220, USA

**Keywords:** type 2 myocardial infarction, coronary angiography, factors associated with utilization, systematic review, invasive management, myocardial injury

## Abstract

**Background/Objectives:** Type 2 myocardial infarction (T2MI) results from a myocardial oxygen supply–demand imbalance without acute coronary atherothrombosis, and the role of invasive coronary angiography in its management is undefined. Prior literature has largely compared T2MI to type 1 myocardial infarction (T1MI) rather than examining angiography selection within T2MI itself. This systematic review with narrative synthesis identified and synthesized factors associated with angiography utilization among T2MI patients, distinguishing adjusted from unadjusted evidence. **Methods:** PubMed, Embase, and ClinicalTrials.gov were searched for studies reporting patient or clinical characteristics associated with undergoing invasive coronary angiography among patients with T2MI, identified via multivariable regression, univariate comparison, or stratified baseline tables. Studies using angiography as an inclusion criterion, an event-risk outcome, or a randomized treatment assignment, or comparing T2MI to T1MI as groups without addressing within-T2MI factors, were excluded. This review was conducted in accordance with PRISMA 2020 guidance. Given clinical and methodological heterogeneity and no common effect-size metric across studies, findings were synthesized narratively without quantitative pooling. **Results:** Three studies met inclusion criteria (two large US administrative-data cohorts, *n* = 268,850 and *n* = 18,606 T2MI; one French two-center cohort, *n* = 224). Younger age was independently associated with greater odds of angiography in the largest cohort reporting age-specific estimates, with a monotonic decline in angiography utilization across advancing age strata; a second cohort’s unadjusted group comparison was directionally concordant, but its own multivariable estimate for age was internally inconsistent and is not treated as confirmatory. Chronic ischemic heart disease and heart failure were independently associated with higher odds of angiography, while chronic kidney disease, chronic obstructive pulmonary disease, peripheral vascular disease, and atrial fibrillation were independently associated with lower odds. Commercial insurance and Midwest or South United States residence were independently associated with higher odds in the single study assessing these factors. Cardiology consultation showed conflicting directions of adjusted association between the two studies reporting it. **Conclusions:** Selection for invasive angiography in T2MI is heterogeneous and inconsistently studied. Age was the only factor with cross-study directional support, and even that lacked confirmed adjusted concordance. Other factors were supported by single studies or conflicting findings, and no included study reported the bedside clinical variables plausibly driving real-time decisions. Certainty was very low for every factor examined (modified GRADE), underscoring the need for prospective study of angiography selection in T2MI.

## 1. Introduction

Type 2 myocardial infarction (T2MI) is defined by the Fourth Universal Definition of Myocardial Infarction as myocardial injury with evidence of ischemia due to an imbalance between myocardial oxygen supply and demand, in the absence of acute atherothrombotic plaque disruption [1]. This absence refers specifically to acute coronary atherothrombosis at the time of the event; patients with T2MI may nonetheless have extensive stable obstructive coronary artery disease, and “absence of unstable coronary artery disease” should not be read as absence of coronary artery disease itself.

This terminology has since evolved. While this review, and every study within it, was conducted under the Fourth Universal Definition’s five-category classification (type 1–5), the Fifth Universal Definition of Myocardial Infarction, published in August 2026, replaced this scheme with three clinical categories, primary, secondary, and procedure-related myocardial infarction, under which the entity previously termed T2MI is now designated secondary myocardial infarction [2]. All three studies included in this review were published before this change (2022–2025) and used “type 2 myocardial infarction” terminology and Fourth Universal Definition criteria throughout; this review retains that terminology for consistency with the course literature rather than retrospectively relabeling it. Diagnostic criteria also differ: The Fifth Universal Definition states that secondary myocardial infarction “cannot be reliably established using symptoms or signs of myocardial ischemia and cardiac troponin levels alone” and calls for additional clinical assessment and, where feasible, cardiac imaging [2], whereas the included studies relied on administrative coding or retrospective troponin/ECG-based adjudication without imaging confirmation (Table 1). Findings here should therefore be treated as informative but not directly transposable to a population defined under the Fifth Universal Definition (Section 4.3).

Unlike T1MI, for which invasive coronary angiography and revascularization are guideline-directed, no comparable consensus exists for T2MI. Reported rates of invasive coronary angiography in T2MI cohorts range widely, from approximately 11% in a large United States national sample to over one-third in smaller cohorts [3,4], reflecting substantial uncertainty in practice.

Prior literature in this space has predominantly compared T2MI to T1MI on baseline characteristics, management intensity, and outcomes, or examined angiography as a protocol-mandated exposure within a defined research cohort. Comparatively little work has examined angiography as itself the outcome of interest—that is, which patient and clinical characteristics are associated with selection for invasive evaluation among patients already diagnosed with T2MI. This distinction matters clinically, since understanding current selection patterns is a prerequisite for the prospective trials needed to test a more standardized invasive approach.

The objective of this systematic review with narrative synthesis was to identify and synthesize the existing literature on patient- and clinical-level factors associated with invasive coronary angiography specifically within T2MI cohorts, following PRISMA 2020 reporting guidance [6]. This review characterizes which factors are statistically associated with current angi-ography utilization as observed in practice; it does not identify which patients would derive clinical benefit from invasive evaluation, a distinct question that current observational evidence is not positioned to answer (Section 4.2).

## 2. Materials and Methods

### 2.1. Protocol and Registration

A protocol was developed before study screening but was not prospectively registered in a public repository. A search of PROSPERO identified no existing registration addressing this specific question; two related but distinguishable protocols were identified (CRD42021237746, CRD42022314132), neither of which frames angiography as the outcome of interest within T2MI. Because data extraction had already been completed by the time registration was considered, prospective registration on PROSPERO was no longer possible, as PROSPERO accepts protocols only prior to the start of extraction. The original protocol has instead been deposited on the Open Science Framework (OSF) with a clearly dated retrospective posting, consistent with a transparent, non-prospective disclosure of the review’s methods.

The registered protocol is available at https://doi.org/10.17605/OSF.IO/RA3D7 (Open Science Framework).

### 2.2. Eligibility Criteria

Studies were eligible if they reported patient demographic, clinical, or system-level characteristics associated with undergoing invasive coronary angiography among patients with an index diagnosis of T2MI. Eligible evidence types were multivariable regression, univariate group comparison, or a stratified baseline characteristics table comparing patients who did versus did not undergo angiography. These three evidence types differ substantially in strength: an adjusted independent association from a multivariable model is not equivalent to an unadjusted difference between groups. Each extracted factor was therefore labeled by evidence type (adjusted vs. unadjusted) at extraction, and this distinction is carried through the Results tables and Discussion rather than treating all reported associations as interchangeable “predictors.”

Studies were excluded on any of four grounds: angiography or another invasive procedure was a protocol-mandated inclusion criterion or exposure rather than an outcome; the study outcome was a downstream clinical event or mortality rather than angiography utilization itself; angiography was assigned by randomization as a trial arm; or the study compared T1MI to T2MI as groups without separately reporting within-T2MI factors associated with angiography. Conference abstracts, preprints, and other non-peer-reviewed or unpublished study reports were not eligible; only peer-reviewed, full-text journal articles and registered trial records (ClinicalTrials.gov) were included. Publication type was assessed during title/abstract screening (Section 2.4) rather than tracked as a separate full-text exclusion category, since no record reaching full-text review was excluded for this reason (Supplementary Table S1).

### 2.3. Information Sources and Search Strategy

PubMed, Embase, and ClinicalTrials.gov were searched from 1 January 2012 to 16 July 2026, restricted to English-language publications, supplemented by citation mining of included and closely related excluded studies. The 1 January 2012 start date was chosen to align with publication of the Third Universal Definition of Myocardial Infarction, which established the currently used framework distinguishing type 1 and type 2 MI in its modern, refined form [7]. The search was updated on 23 September 2026 to reflect literature published since the original search date, adding “secondary myocardial infarction” to the search terms following the publication of the Fifth Universal Definition of Myocardial Infarction (Section 1) [2]; the updated search and its results are reported in Section 3.1.

The following search string was used for PubMed and Embase:

((“myocardial infarction” AND (“type 2” OR “type II” OR “T2MI”)) OR “T2MI” OR “type 2 myocardial infarction” OR “type II myocardial infarction” OR “myocardial oxygen supply-demand mismatch” OR “supply-demand mismatch myocardial infarction”) AND (“coronary angiography” OR “invasive management” OR “invasive coronary angiography” OR “cardiac catheterization” OR “revascularization” OR “percutaneous coronary intervention” OR “PCI” OR “conservative management” OR “LHC”), limited to 1 January 2012–16 July 2026. The updated search (Section 3.1) added “secondary myocardial infarction” to the first term group; the complete updated string is given in Supplementary Table S2.

This search used free-text keywords only, without MeSH (PubMed) or Emtree (Embase) controlled vocabulary terms, and was limited to English-language publications from 1 January 2012 to 16 July 2026. Adding the controlled-vocabulary headings for myocardial infarction (MeSH “Myocardial Infarction”, D009203; Emtree “heart infarction”) was tested but not adopted. Neither term, not any narrower term under either heading, distinguishes type 2 from other myocardial infarction subtypes.

For ClinicalTrials.gov, records were identified using free-text terms (“type 2 myocardial infarction” OR T2MI OR “type II myocardial infarction” OR “secondary myocardial infarction”), without restricting to the platform’s structured “Condition/disease” field, which an earlier attempt using that field was found to under-capture (Section 3.1).

The Embase search was executed via embase.com. Both the PubMed and Embase searches were run on 16 July 2026.

### 2.4. Study Selection

Two reviewers (Ngoc Thai Kieu and Mays Tawayha) independently screened titles and abstracts and subsequently assessed potentially eligible full-text reports. Disagreements were resolved through discussion and, when necessary, adjudication by a third reviewer (Manoj Sharma). A formal inter-reviewer agreement statistic (e.g., Cohen’s kappa) was not calculated for either screening stage; agreement was managed through discussion and adjudication rather than quantified. Records were screened in a two-stage process (title/abstract, then full text), tracked using a dedicated screening log, exclusion tally, and overlap tracker. A total of 5585 records were identified through database searching (PubMed, *n* = 1348; Embase, *n* = 4219; ClinicalTrials.gov, *n* = 18, using the corrected free-text strategy—Section 2.3). Before screening, 1239 duplicate records were identified and removed by a single reviewer (Ngoc Thai Kieu) through manual comparison of title and author within Rayyan rather than by automated matching, leaving 4346 records for title and abstract screening. Following title/abstract screening, 4333 records were excluded as clearly not relevant, leaving 13 reports identified through the database search that were sought for full-text retrieval. An additional 5 records were identified through citation searching of included and closely related excluded studies; these were sought for retrieval directly, without title/abstract screening, since citation searching is targeted rather than exhaustive. All 18 were retrieved and assessed for eligibility. Of these, 15 were excluded at full-text review for the reasons summarized in Section 3.1 and detailed in Supplementary Table S1, and 3 studies met full inclusion criteria.

### 2.5. Data Extraction

Data extraction and risk-of-bias assessments were performed independently by two reviewers using standardized forms, with discrepancies resolved through discussion; as with screening (Section 2.4), no formal inter-reviewer agreement statistic was calculated for extraction or QUIPS ratings. Data were extracted using a factor-level master extraction template, capturing study design, data source, population, angiography outcome definition, and each reported factor with its associated effect estimate, comparison group, evidence type (adjusted vs. unadjusted, per Section 2.2), and precision (confidence interval or *p*-value) where available.

Where a study did not report a given factor, effect estimate, confidence interval, or reference category, this was recorded as not reported/not available rather than estimated or imputed by the review authors; no imputation was performed.

One exception applies to Fournier et al.’s multivariable age estimate, whose OR and 95% CI were not printed in the source text and existed only as a point and error bars in a published forest plot (Fournier et al.’s Figure 4); values were digitized by measuring pixel positions against the plot’s axis tick marks and converting them via its log-linear scale. This estimate is labeled as digitized wherever presented (Section 3.3; Table 2 and was excluded from quantitative pooling assessment (Section 2.7).

### 2.6. Risk of Bias Assessment

QUIPS [8] was selected over exposure-focused tools such as ROBINS-I because QUIPS is structured for studies examining one or more candidate factors’ association with an outcome across a defined cohort—the design used by all three included studies, each of which examined multiple candidate factors rather than a single pre-specified exposure. This structure is analogous to prognostic factor research, since the included studies evaluated associations between baseline patient- and system-level factors and a subsequent clinical management outcome. Domains were interpreted in the context of angiography utilization rather than prognosis; “prognostic factor measurement” is used here to refer to measurement of the utilization-associated factor rather than a prognostic marker. To limit subjectivity in domain-level judgments, both reviewers applied a written decision-rule rubric, adapted from the standard QUIPS guidance to this review’s utilization outcome, independently before reconciling ratings by consensus discussion. The full rubric and the resulting consensus judgments for each domain and study are provided in Supplementary Table S4. Domain-level ratings (low, moderate, or high risk of bias) for each included study are presented in Table 1 (Section 3.3).

### 2.7. Data Synthesis

The effect measure extracted and reported for each factor was the odds ratio (adjusted or unadjusted, per Section 2.2), as reported by the original study; no effect measure was calculated or converted by the review authors, with the single exception described in Section 2.5 (Fournier et al.’s digitized age estimate), which was excluded from quantitative use.

A narrative synthesis was performed as the analytic approach for this review. The three included studies differed materially in data source (a national weighted hospital-discharge administrative database, a national commercial/Medicare Advantage claims database, and a single-country two-center cohort), population, outcome definition, and covariate sets, and study populations are not summed into a single overall sample anywhere in this review given these differences, and no factor was reported using a common effect-size metric by more than one study. Age came closest to a factor reported by two studies but was not quantitatively pooled: the larger study reported a multivariable-adjusted categorical odds ratio across age strata, while the second study’s only usable age data were an unadjusted mean comparison with a *p*-value and no reported effect size, and its own multivariable estimate for age was internally inconsistent with its univariate finding. In the absence of a shared metric, and given that one of the two data points was unadjusted, this discrepancy is presented narratively rather than forced into a pooled estimate. No quantitative meta-analysis was therefore performed for any factor.

Because no quantitative synthesis was performed, statistical exploration of heterogeneity, sensitivity analyses, formal assessment of reporting bias (e.g., funnel-plot-based methods), were not conducted. These methods generally require pooled or multi-study quantitative estimates, which were not available for any factor in this review, their omission is noted as a methodological limitation in Section 4.3. A certainty-of-evidence assessment does not require pooling, however, and was performed for every factor domain using a modified GRADE approach adapted for narrative synthesis, following published guidance for rating certainty in the absence of a single pooled estimate of effect [9]. Following standard GRADE practice for observational evidence, each factor began at Low certainty. Certainty was then rated down one level for each domain (risk of bias, inconsistency, indirectness, imprecision) judged “serious,” and two levels for a domain judged “very serious”; inconsistency was treated as not estimable where only one study reported a factor. Publication bias was not assessed given the small number of included studies. Ratings and their rationale are presented in Table 3 (Section 3.5).

### 2.8. Use of Generative Artificial Intelligence

Generative AI tools (ChatGPT-5 (OpenAI) and Claude Sonnet 5 (Anthropic)) were used during the preparation of this manuscript for literature synthesis support and manuscript drafting, as detailed in the Acknowledgments. All study eligibility decisions, data extraction, risk-of-bias judgments, certainty-of-evidence ratings, and interpretation of findings were performed by the human authors, as described in Section 2.4, Section 2.5, Section 2.6 and Section 2.7.

## 3. Results

### 3.1. Study Selection

**Figure 1:**
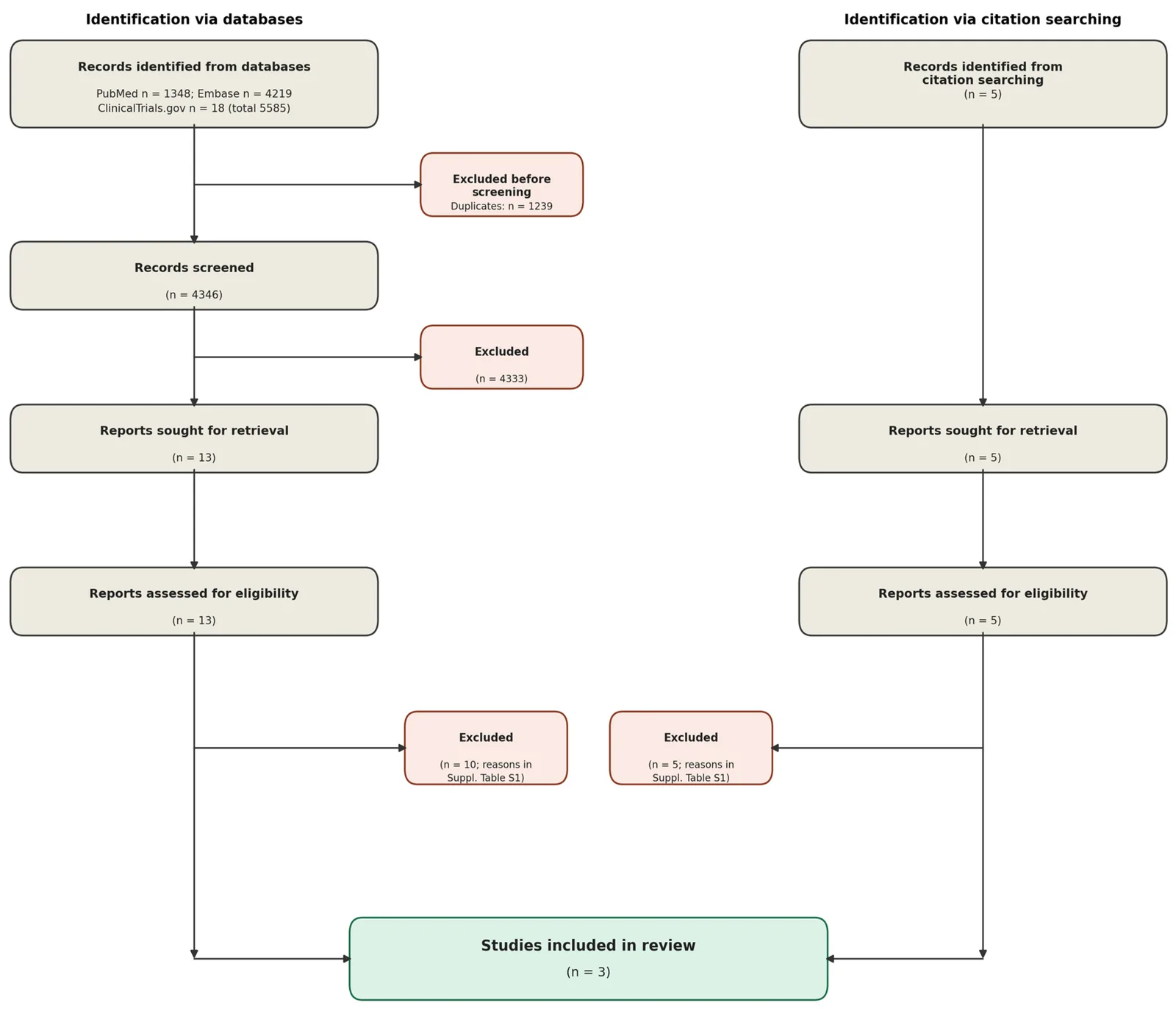
PRISMA 2020 flow diagram of study identification, screening, and inclusion.

Three studies met full inclusion criteria (Figure 1): Smilowitz et al. [3], Fournier et al. [4], and Goel et al. [5], all identified through the database search. Fifteen additional candidates were excluded at full-text review: ten identified via database search and five identified via citation searching (Tripathi et al., 2021; Smilowitz et al., 2016; Coscia et al., 2022; Chapman et al., 2018; Eggers et al., 2023 [SWEDEHEART]) that were not returned by the PubMed/Embase/ClinicalTrials.gov search string despite falling within the same topical scope—consistent with the free-text-only search strategy’s limited sensitivity (Section 4.3). These fell into five categories: protocol-mandated imaging or procedure, where angiography was an inclusion criterion rather than a clinician-selected outcome (*n* = 2: McCarthy et al., 2023 [DEFINE-MI]; Bularga et al., 2022 [DEMAND-MI]); randomized assignment of invasive management as a trial intervention (*n* = 1: Taggart et al., 2025 [TARGET-Type 2]); an event-risk or prognostic outcome rather than angiography utilization (*n* = 3: Tripathi et al., 2021; Coscia et al., 2022; Raphael et al., 2020); T1MI versus T2MI (or T2MI versus non-MI myocardial injury) group comparisons that did not report within-T2MI factors (*n* = 8: Wereski et al., 2022; Smilowitz et al., 2016; the Sheba Medical Center cohort study; McCarthy et al., 2021; Chapman et al., 2018; Stein et al., 2014; Baron et al., 2015 [TOTAL-AMI]; Eggers et al., 2023 [SWEDEHEART]); and one existing meta-analysis that, per its own stated limitations, could not perform multivariable analysis of associated factors due to lack of individual patient data (White et al., 2022). The complete list of excluded studies with study-specific reasons is provided in Supplementary Table S1.

Following peer review, the search was updated on 23 September 2026 to reflect literature published since the original search date and to add “secondary myocardial infarction” following publication of the Fifth Universal Definition of Myocardial Infarction [2] (Section 2.3). The updated search identified 43 new PubMed records and 91 new Embase records (89 under the original terms plus 2 attributable specifically to “secondary myocardial infarction”); no records dated within the update period were identified in ClinicalTrials.gov. Separately, the original ClinicalTrials.gov strategy was found to under-capture eligible registrations because of its reliance on the platform’s structured “Condition/disease” field rather than free-text matching; a corrected free-text search of the original date range (1 January 2012–16 July 2026) identified 18 records rather than the 14 originally reported. All 134 update-period records (43 PubMed; 91 Embase) and the corrected ClinicalTrials.gov set were screened at title/abstract against the eligibility criteria in Section 2.2; none met inclusion criteria, and the three studies identified in the original search remain the complete eligible evidence base for this review.

### 3.2. Study Characteristics

The three included studies do not report an identical angiography outcome. Smilowitz et al. [3] defined the outcome as in-hospital invasive coronary angiography with or without revascularization, ascertained within a single index hospitalization. Fournier et al. [4] defined it as in-hospital invasive coronary exploration, also within a single index hospitalization, without distinguishing whether revascularization followed. Goel et al. [5] defined it as coronary angiography occurring either during the index admission or at any point within 6 months post-discharge, a combined, non-time-ordered window that mixes acute and post-acute care. These are related but non-equivalent constructs: they differ in whether revascularization is bundled into the outcome and in whether the assessment window is limited to the index hospitalization or extends 6 months beyond it. This matters directly for two of the cross-study comparisons in Table 3: the age comparison between Goel et al. and Fournier et al. (Section 3.4) pairs a 6-month combined-window outcome with an in-hospital-only outcome, and the cardiology-consultation discrepancy (Section Unresolved Cross-Study Discrepancy: Cardiology Consultation) arises between the same two non-equivalent outcome definitions. Both comparisons are presented as directional patterns rather than evidence of a single underlying construct, and neither should be read as though the three studies measured the same event on the same clock.

### 3.3. Risk of Bias

Risk of bias was assessed across the six QUIPS domains for each included study; ratings are summarized in Table 2, with the rationale for each rating given in Supplementary Table S5.

The most consequential risk-of-bias finding is specific rather than general: Fournier et al.’s multivariable model reports that age below 75 years independently associated with the absence of invasive exploration (Table 3)—the opposite direction from the same study’s own univariate comparison and from Goel et al. Because the published report did not permit resolution of this discrepancy, the multivariable age estimate was considered uninterpretable for the purposes of cross-study synthesis (Section 3.4) and was not used in the assessment of whether age could be quantitatively pooled across studies (Section 2.7). Beyond this specific issue, Fournier et al. carries the highest overall risk of bias among the three included studies, driven by its restriction to patients with no pre-existing cardiac history at only two centers (study participation), a small event count that the authors themselves note constrained model stability (study confounding), and adjudication by a single unblinded reviewer (prognostic factor measurement). Smilowitz et al. and Goel et al. are both large, well-adjusted claims-based analyses with lower overall risk of bias, though both share the general limitation of administrative/claims data lacking clinical granularity (laboratory values, imaging findings, symptom severity), and Goel et al.’s exclusion of patients who died, were discharged to hospice, or lacked continuous enrollment may selectively remove the most acutely ill patients from its predictor analysis.

### 3.4. Narrative Synthesis of Factors Associated with Angiography Utilization

Findings are organized by factor domain in Table 3, grouped into three sections: adjusted findings with full effect estimates reported, adjusted findings for which the source study reported only a *p*-value without a point estimate or confidence interval, and adjusted findings that are internally discordant or unresolved across studies and should be interpreted with particular caution; which studies reported each factor and whether the evidence was adjusted or unadjusted is noted in the same column. Because baseline patient characteristics, clinical presentation, hospital/payer context, and care-process events occupy different temporal and causal roles relative to angiography selection, each factor is additionally classified into one of five categories: baseline patient factors, presenting clinical factors, hospital and payer factors, concurrent care-process factors, and post-discharge factors with uncertain temporal relationship to the outcome. This classification is descriptive rather than causal—it clarifies when in the care pathway each factor was measured, not whether it caused angiography selection.

#### Unresolved Cross-Study Discrepancy: Cardiology Consultation

The direction of association between cardiology consultation and angiography utilization conflicted between the two studies reporting it. In Goel et al. [5], consultation with a cardiologist at the index admission was associated with lower odds of angiography (adjusted OR 0.72, 95% CI 0.57–0.92) within the 6-month post-discharge assessment window, while post-discharge cardiology follow-up was associated with higher odds (adjusted OR 2.26, 95% CI 1.89–2.71) over the same window. Because Goel et al. measured angiography, index consultation, and post-discharge follow-up within a shared 6-month window rather than a strictly sequential design, the temporal ordering between a post-discharge cardiology visit and angiography could not be established from the reported analysis; post-discharge cardiology follow-up was associated with angiography utilization, but this association should not be read as directional or causal. In Fournier et al. [4], a cardiology consultation request was associated with higher odds of invasive exploration. These two findings are not necessarily contradictory, they may reflect different constructs and different points in the care pathway, but the available data do not permit reconciliation, and this is presented as an open question rather than resolved in either direction. Resolving it would require a study that records, for each patient, the timestamp and clinical content of any cardiology consultation relative to the timestamp of angiography (rather than a shared assessment window covering both), so that consultations occurring before versus after the angiography decision can be distinguished, along with the reason the consultation was requested (e.g., troponin elevation versus a specific ischemic concern); without that temporal and contextual detail, the direction of this association cannot be adjudicated from administrative or retrospective chart data of the kind available here.

### 3.5. Certainty of Evidence

Table 4 presents a modified GRADE certainty rating for each factor domain in Table 3, following the approach described in Section 2.7. That every factor reaches the same Very Low rating reflects the evidence base’s uniform reliance on one or two administrative-data studies per factor, rather than an artifact of how the framework was applied. Every factor in this review starts from the Low-certainty baseline used for observational evidence and is downgraded further where risk of bias, inconsistency, indirectness, or imprecision was judged serious or very serious; because Low is already the second lowest of the four GRADE certainty levels, a single further serious limitation is sufficient to reach Very Low, the floor of the scale. Every factor examined in this review reaches that floor: the review question has not yet been addressed by evidence capable of supporting more than very-low-certainty conclusions, for reasons that differ somewhat by factor but recur across all nine domains—reliance on one or two administrative-data studies, non-equivalent outcome or exposure constructs across studies, and, for two factors, an internal or cross-study contradiction that could not be resolved from the published reports.

Rationale for each rating: age was downgraded for risk of bias because Fournier et al. carried high-risk QUIPS ratings in several domains, for inconsistency because Fournier’s own adjusted estimate reversed its unadjusted finding, and for indirectness because Goel’s 6-month combined-window outcome and Fournier’s in-hospital-only outcome are non-equivalent constructs (Section 3.2). Insurance/payer status and geographic region/hospital characteristics were downgraded for risk of bias on the basis of Smilowitz et al.’s Moderate ratings for prognostic factor measurement and study confounding. Comorbidity/cardiac disease burden and sex/race-ethnicity (both Goel-only) were downgraded for risk of bias on similar grounds and for indirectness owing to the combined index-admission-plus-6-month outcome window. Cardiology consultation (index) was downgraded for inconsistency because the two studies reported directly conflicting directions of association that could not be reconciled (Section Unresolved Cross-Study Discrepancy: Cardiology Consultation), and for indirectness because the two studies measured different care-process constructs. Cardiology consultation (post-discharge) was downgraded for indirectness at the “very serious” level because the combined assessment window precluded establishing whether the exposure preceded the outcome at all. Clinical presentation/LVEF and family history of vascular disease (both Fournier-only) were downgraded for risk of bias at the “very serious” level reflecting Fournier et al.’s high-risk ratings for study participation, prognostic factor measurement, and confounding, and for imprecision because the source study reported only *p*-values without point estimates or confidence intervals.

## 4. Discussion

This systematic review identified only three studies that examined factors associated with invasive coronary angiography as an outcome within T2MI cohorts specifically, despite a much larger body of literature on T2MI overall. That scarcity is itself a principal finding, consistent with the literature gap described in Section 1. Across the three studies that did address this question, younger age was the only factor showing a directionally similar association in more than one study, although adjusted confirmation was available from only one cohort. Several cardiac comorbidities were independently associated with angiography utilization in that same single large cohort. Insurance status, geographic and hospital-level variation, and the timing and directionality of cardiology involvement remain supported by only a single study each, or by directly conflicting findings.

### 4.1. Interpretation in the Context of Broader T2MI Literature

The age gradient identified here is consistent with a more general pattern in acute coronary syndrome care, in which older patients are less likely to be referred for invasive evaluation even after adjustment for comorbidity, frailty concerns, and perceived procedural risk [10]. In T2MI specifically, this raises a particular concern: because T2MI itself occurs disproportionately in older, multimorbid patients, an age-based reduction in angiography referral compounds with a population that is already less likely to be investigated for reasons related to its precipitating illness (sepsis, respiratory failure, and similar systemic conditions), rather than reasons related to coronary anatomy. Obstructive coronary disease is genuinely prevalent in T2MI, reported at 64% in an angiographic series of T2MI patients outside the scope of this review’s eligibility criteria [11]; that figure is cited here only as background context on T2MI’s coronary disease burden generally, not as age-stratified evidence bearing on the undertreatment question itself. None of the three included studies reported angiographic findings or downstream revascularization decisions stratified by age, and none adequately captured illness severity, frailty, or procedural contraindications that could plausibly explain a lower angiography rate in older patients on clinical grounds rather than through undertreatment. Residual confounding by these unmeasured factors cannot be excluded.

The regional and payer-based variation reported by Smilowitz et al. [3], in particular, raises the possibility that angiography selection in T2MI is driven as much by structural and access-related factors as by clinical risk stratification—a pattern previously described in T1MI but not, prior to this literature, systematically documented in T2MI. Commercially insured (18.2%) and self-pay patients (17.3%) underwent angiography at roughly twice unadjusted rate observed for Medicare patients (9.6%); Medicaid patients underwent angiography at an intermediate rate (12.9%). In the multivariable model, for which Medicare (not Medicaid) was the reference category, commercial insurance remained independently associated with higher odds of angiography (aOR 1.39, 95% CI 1.27–1.52) and Medicaid with lower odds (aOR 0.86, 95% CI 0.76–0.96) versus Medicare. Hospital-level variation persisted independent of patient case mix. These associations may reflect differences in access, referral patterns, hospital resources, residual clinical confounding, or other unmeasured structural factors; the included data cannot determine the relative contributions of these mechanisms. This is a materially different problem from the age-based gradient described above: it is not obviously traceable to a single defensible clinical rationale, and it mirrors disparities in invasive management that have been documented in T1MI for over two decades without full resolution [12,13,14].

The unresolved discrepancy in the direction of the cardiology-consultation factor (Section Unresolved Cross-Study Discrepancy: Cardiology Consultation) most likely reflects that the two studies measured different constructs and different points in the care pathway: an index-admission consultation captured in a claims database is not necessarily the same clinical event as a consultation request recorded in a two-center cohort, and neither study reported enough detail about timing or content to establish whether the two represent the same decision point. Rather than adjudicate between the two directions, this review treats the discrepancy as evidence that consultation timing and construct must be defined consistently before this factor can be meaningfully compared across studies.

### 4.2. Clinical and Research Implications

For clinicians, the practical implication is that current angiography selection in T2MI is not obviously anchored to a consistent or validated risk model. Age, the factor with the clearest cross-study directional support, is a marker of frailty and competing mortality risk as much as it is a marker of coronary disease likelihood, and none of the included studies linked its factor set to angiographic yield or to a validated pretest probability tool. In the absence of validated selection tools, these findings reinforce the need for individualized decision-making rather than reliance on demographic, payer, or regional factors alone.

A principal finding of this review is not any single associated factor but an absence: none of the three included studies reported the bedside clinical variables that plausibly drive real-time angiography decisions, as distinct from the demographic and administrative factors this review was able to synthesize (Table 5). This gap is a central finding in its own right, not merely a limitation of the available evidence, because it means the factor set this review characterizes and the factor set that actually informs bedside decision-making may barely overlap.

For the research community, this review’s central contribution is negative: it demonstrates that the evidence base for angiography selection in T2MI is too sparse and too methodologically heterogeneous to support quantitative synthesis, amid a rapidly growing volume of T2MI outcomes literature. Future observational studies in this area would substantially increase their value to the field by reporting a common minimum factor set using comparable, adjusted effect-size metrics: at minimum, age as a continuous or standardized categorical variable, comorbidity burden, and cardiology involvement explicitly defined by timing. Doing so would keep future reviews from being left, as this one was, unable to pool even the single factor with the most cross-study support. Future studies should also adopt the Fifth Universal Definition’s “secondary myocardial infarction” terminology and imaging-inclusive criteria [2], while still reporting the legacy T2MI label during the transition period so results remain interpretable against this review’s evidence base. Prospective, protocolized study of which patients derive benefit from invasive evaluation is the more direct route to resolving the underlying clinical question. The ACT-2 randomized trial was designed to evaluate the clinical and economic effects of early coronary investigation in patients with myocardial injury or T2MI [15]. More recent work, including the TARGET-Type 2 pilot randomized trial and a contemporary review highlighting the diagnostic value of coronary imaging and the risk of T2MI misclassification, further underscores that this is an active area of investigation [16,17]. In the interim, more consistently reported observational data would meaningfully improve the field’s ability to characterize current practice.

### 4.3. Limitations

- This review’s evidence base predates a terminology and diagnostic-criteria change: The Fifth Universal Definition of Myocardial Infarction (August 2026) now terms T2MI “secondary myocardial infarction” and requires imaging confirmation that none of the three included studies applied (Section 1). This review’s findings should therefore be read as evidence about T2MI as historically defined and ascertained, not as evidence already validated against the current diagnostic framework.
- Only three studies met eligibility criteria, and each using a different data source, population, covariate set, and angiography outcome definition (Section 3.2), which precluded meaningful quantitative pooling for any factor. Even where two studies reported the same factor domain with adjusted estimates from both (e.g., age), their adjustment sets differed substantially in size and composition (Goel’s model adjusts for approximately 20 covariates versus a smaller set in Fournier). The term “consistency” in Table 3 therefore reflects directional agreement only, not comparability of adjusted effect magnitude. As a narrative synthesis, this review cannot provide a pooled effect estimate for any factor, and conclusions about the relative strength of associations are qualitative.
- Two of the three included studies relied on administrative coding (a weighted hospital-discharge database and a commercial/Medicare Advantage claims database, respectively), rather than independent clinical adjudication. This is subject to misclassification of both the T2MI diagnosis itself (including confusion with T1MI or acute nonischemic myocardial injury) and the invasive procedures performed, and such misclassification may have influenced both cohort composition and observed associations in these two studies.
- The single study reporting insurance and geographic factors is a single national cross-sectional sample from a single calendar year (2018), and these associations have not been externally replicated.
- Age, the factor with the strongest cross-study support, is nonetheless supported by an adjusted estimate from only one study; the second study’s age data were an unadjusted comparison, internally inconsistent with its own multivariable text, and could not be used as an independent adjusted confirmation.
- Cardiology consultation is reported using non-equivalent constructs across studies (timing and nature of consultation are defined differently), limiting direct comparison.
- Goel et al.’s predictor analysis excluded patients who died in-hospital, were discharged to hospice, or lacked 6 months of continuous insurance enrollment; this may selectively remove the most acutely ill patients—plausibly those least likely to undergo angiography—and could bias that study’s factor estimates toward a healthier subpopulation. The same study defines its comorbidity covariates as “present within 6 months of index admission” without specifying whether ascertainment preceded the index event or could overlap with the index-admission-plus-6-month-postdischarge window used to define the procedure/consultation outcome itself (Table 3). If the two windows overlap, a comorbidity coded after a procedure or consultation could be misread as a predictor of it, which affects how those odds ratios should be interpreted.
- The search was limited to English-language records from 1 January 2012 onward (aligned with publication of the Third Universal Definition of Myocardial Infarction); relevant studies published before this refinement of the MI classification system, or in other languages, could still have been missed.
- The original ClinicalTrials.gov search relied on the platform’s structured “Condition/disease” field, which was subsequently found to under-capture eligible registrations relative to free-text matching of the same terms; a corrected free-text search of the identical date range found 18 records rather than the 14 originally reported (Section 3.1). This discrepancy was identified only at the search-update stage, and the same field-matching limitation could in principle have affected sensitivity during the original screening period as well.
- The database search used free-text keywords only, without MeSH (PubMed) or Emtree (Embase) controlled vocabulary; adding the only available MeSH/Emtree headings for myocardial infarction was tested and rejected, since it increased PubMed and Embase result counts by more than an order of magnitude without adding type-2-specific discrimination (Section 2.3). Five of the 18 full-text-assessed reports in this review (including all citation-search-only exclusions; Section 3.1) were identified only through citation searching rather than by the search string itself, indicating the database search alone missed a meaningful share of topically relevant literature; citation searching was relied on to partially offset this sensitivity gap.
- The absence of bedside clinical variables from the available evidence (Table 5; Section 4.2) is itself a limitation of what this review can conclude: the factor set characterized here should not be read as a complete or clinically sufficient account of what drives angiography selection in practice.
- No formal inter-reviewer agreement statistic was calculated at any review stage (Section 2.4, Section 2.5 and Section 2.6), so the reproducibility of individual screening and rating decisions cannot be reported numerically.
- Most significantly, this review’s protocol was not prospectively registered. The protocol was developed before screening began, but formal registration was only considered after data extraction had already been completed. At that point, prospective registration on PROSPERO was no longer possible, since PROSPERO accepts registrations only prior to the start of extraction (Section 2.1). The protocol was therefore deposited retrospectively, with a clearly dated posting, on the Open Science Framework. This is a material limitation rather than a formality. Retrospective registration cannot rule out the possibility that eligibility criteria, factor selection, or analytic decisions were, even unintentionally, shaped by preliminary findings already in hand at the time the protocol was written down, and it removes the independent, dated record that prospective registration is intended to provide. Readers should weigh the review’s conclusions with this limitation in mind.

## 5. Conclusions

Evidence regarding factors associated with invasive coronary angiography use in T2MI is sparse and methodologically heterogeneous. Younger age showed a directionally similar association in two studies, although concordant adjusted evidence was available from only one cohort; the second cohort’s own adjusted model found the opposite direction, an internal inconsistency the published report did not permit resolving (Section 3.3). Associations involving comorbidity, insurance, geography, hospital characteristics, and cardiology involvement were either supported by one study or defined inconsistently across studies. A modified GRADE assessment (Section 3.5) rated certainty as very low for every factor examined, reflecting reliance on one or two administrative-data studies per factor, non-equivalent outcome and exposure definitions across studies, and, for two factors, an unresolved internal or cross-study contradiction. Current evidence therefore cannot identify a validated set of factors for selecting patients with T2MI for invasive evaluation. Prospective studies incorporating clinical presentation, ischemic findings, coronary anatomy, procedural risk, and patient-centered outcomes are needed, including trials such as ACT-2 [15].

## Figures and Tables

**Table 1:** Summarizes the design, setting, and factors examined by each of the three included studies.

Study	Design, Data Source and Setting	T2MI Diagnosed, *n*/Analytic Sample, *n*	Assessment Window	Angiography Outcome Definition	Factors Examined (Within-T2MI; Adjustment Status Noted)	T2MI Ascertainment
Smilowitz et al. [3]	Cross-sectional; NIS 2018 (weighted US hospital-discharge sample).	268,850 diagnosed (same as analytic sample).	Single index hospitalization	In-hospital invasive coronary angiography ± revascularization (11.2% overall)	Insurance payer, region, hospital size/teaching status (all adjusted; age adjusted for but not reported).	ICD-10-CM I21.A1; no independent adjudication; Fourth UDMI-aligned coding.
Fournier et al. [4]	Retrospective cohort; 2 French centers (±cath lab).	224 diagnosed (same as analytic sample).	Single index hospitalization	In-hospital invasive coronary exploration (34.8% overall)	Family history, cardiology consultation, LVEF, NSTEMI/STEMI (all adjusted); age (unadjusted only—adjusted estimate discordant, not used; Section 3.3).	Chart review, adjudicated a posteriori by a single reviewer per ESC/Fourth UDMI criteria plus Saaby et al.’s objective mismatch criteria.
Goel et al. [5]	Retrospective cohort; Optum Clinformatics Data Mart (US commercial/Medicare Advantage claims).	18,606 diagnosed (of 140,344 total MI); 14,176 analytic sample (exclusions detailed in Section 4.3).	Index admission plus 6 months post-discharge, combined into a single non-time-ordered window	Coronary angiography during index admission or within 6 months post-discharge, multivariable-adjusted	Age, chronic ischemic heart disease, CHF, CKD, COPD/asthma, PVD, AFib, region, race, sex, cardiology consultation (index/post-discharge)—all adjusted.	ICD-10-CM I21.A1; no independent adjudication; Fourth UDMI-aligned coding.

NIS: National Inpatient Sample. LVEF: left ventricular ejection fraction. NSTEMI/STEMI: non-ST-elevation/ST-elevation myocardial infarction. CHF: congestive heart failure. COPD: chronic obstructive pulmonary disease. CKD: chronic kidney disease. PVD: peripheral vascular disease. AFib: atrial fibrillation. UDMI: Universal Definition of Myocardial Infarction.

**Table 2:** Risk-of-bias ratings by study and QUIPS domain.

QUIPS Domain	Smilowitz et al. [3]	Fournier et al. [4]	Goel et al. [5]
Study participation	Low	High	Moderate
Study attrition	Low	Low	Moderate
Prognostic factor measurement	Moderate	High	Moderate
Outcome measurement	Low	Moderate	Low
Study confounding	Moderate	High	Moderate
Statistical analysis and reporting	Low	Moderate (age estimate discordant; Section 3.4)	Low

QUIPS: Quality In Prognosis Studies.

**Table 3:** Factors associated with invasive coronary angiography use in type 2 myocardial infarction, grouped by strength of evidence.

Factor Domain	Category	Direction of Association with Angiography	Studies Reporting and Evidence
Adjusted findings with full effect estimates reported			
Insurance/payer status	Hospital and payer factor	Commercial insurance associated with higher odds (aOR 1.39, 95% CI 1.27–1.52) and Medicaid with lower odds (aOR 0.86, 95% CI 0.76–0.96) of invasive management, reference category Medicare.	Smilowitz only (adjusted)
Geographic region/hospital characteristics	Hospital and payer factor	Substantial regional heterogeneity (7.2–13.8% across US census divisions); higher odds in Midwest (aOR 1.27, 95% CI 1.11–1.46) and South (aOR 1.34, 95% CI 1.18–1.53) versus reference category Northeast; higher odds at large and urban teaching hospitals versus small/rural hospitals (exact values in source study).	Smilowitz only (adjusted)
Comorbidity/cardiac disease burden	Baseline patient factor	Chronic ischemic heart disease and CHF/exacerbation associated with higher odds (aOR 2.24, 95% CI 1.96–2.56 and aOR 1.33, 95% CI 1.16–1.52, respectively); chronic kidney disease, COPD/asthma, peripheral vascular disease, and atrial fibrillation/flutter/PSVT/VT associated with lower odds (aOR 0.76, 95% CI 0.68–0.86; aOR 0.75, 95% CI 0.67–0.85; aOR 0.82, 95% CI 0.71–0.95; aOR 0.89, 95% CI 0.79–0.99, respectively). As with the post-discharge cardiology finding (Section Unresolved Cross-Study Discrepancy: Cardiology Consultation), these comorbidities’ ascertainment window may overlap with the outcome-assessment window—see Section 4.3 for detail.	Goel only (adjusted)
Sex, race/ethnicity	Baseline patient factor	No significant association (Black vs. White aOR 0.86, 95% CI crossing 1; female vs. male aOR 0.97, 95% CI crossing 1).	Goel only (adjusted); null finding
Adjusted findings—effect size not fully reported by the source study (*p*-value only)			
Clinical presentation/LVEF	Presenting clinical factor	Preserved LVEF and NSTEMI (vs. STEMI-pattern) presentation associated with lower odds of angiography (multivariable *p* = 0.020 and *p* = 0.020 0.034, respectively; exact aOR and 95% CI not reported in the source study).	Fournier only (adjusted)
Family history of vascular disease	Baseline patient factor	Positive family history associated with higher odds of angiography (multivariable *p* = 0.020 0.006; exact aOR and 95% CI not reported in the source study).	Fournier only (adjusted)
Adjusted findings—internally discordant or unresolved across studies (interpret with particular caution)			
Age	Baseline patient factor	Goel [5]: monotonic decline across age strata vs. reference <65 years (70–74 aOR 0.71, 95% CI 0.59–0.86; 75–79 aOR 0.64, 95% CI 0.53–0.77; 80–84 aOR 0.48, 95% CI 0.39–0.59; 85+ aOR 0.20, 95% CI 0.16–0.25). Fournier’s [3] unadjusted comparison is concordant (70 vs. 78 years, *p* < 0.001), but Fournier’s own multivariable estimate (age < 75 vs. ≥75 years; OR ≈ 0.29, 95% CI ≈ 0.13–0.60, *p* = 0.020 0.001, digitized from Fournier et al.’s Figure 4) reverses this direction and was uninterpretable for synthesis (Section 3.3).	Goel (adjusted) & Fournier (unadjusted only); directionally consistent but not confirmed in >1 adjusted cohort; not reported by Smilowitz
Cardiology consultation (index admission/consultation request)	Concurrent care-process factor	Direction conflicts: Goel [5] found lower odds (aOR 0.72, 95% CI 0.57–0.92); Fournier found higher odds of exploration.	Goel & Fournier (both adjusted); direction conflicts—unresolved (Section Unresolved Cross-Study Discrepancy: Cardiology Consultation)
Cardiology consultation (post-discharge)	Post-discharge factor	Higher odds (aOR 2.26, 95% CI 1.89–2.71) within the same 6-month window as the outcome; temporal order not establishable—see Section Unresolved Cross-Study Discrepancy: Cardiology Consultation.	Goel only (adjusted); not comparable to the index-consultation direction above

aOR: adjusted odds ratio. CI: confidence interval. CHF: congestive heart failure. COPD: chronic obstructive pulmonary disease. PSVT: paroxysmal supraventricular tachycardia. VT: ventricular tachycardia. LVEF: left ventricular ejection fraction. NSTEMI/STEMI: non-ST-elevation/ST-elevation myocardial infarction.

**Table 4:** Certainty of evidence for each factor domain, rated using a modified GRADE approach.

Factor	Risk of Bias	Inconsistency	Indirectness	Imprecision	Certainty
Age	Serious	Serious	Serious	Not serious	Very Low
Insurance/payer status	Serious	Not estimable (single study)	Not serious	Not serious	Very Low
Geographic region/hospital characteristics	Serious	Not estimable (single study)	Not serious	Not serious	Very Low
Comorbidity/cardiac disease burden	Serious	Not estimable (single study)	Serious	Not serious	Very Low
Cardiology consultation (index)	Serious	Very serious	Serious	Not serious	Very Low
Cardiology consultation (post-discharge)	Serious	Not estimable (single study)	Very serious	Not serious	Very Low
Clinical presentation/LVEF	Very serious	Not estimable (single study)	Not serious	Serious	Very Low
Family history of vascular disease	Very serious	Not estimable (single study)	Not serious	Serious	Very Low
Sex, race/ethnicity	Serious	Not estimable (single study)	Serious	Not serious	Very Low

**Table 5:** Factors examined in the included studies versus clinically relevant factors not examined in any included study.

Examined in the Included Studies	Clinically Relevant but Not Examined in Any Included Study
Age Sex, race/ethnicity Insurance/payer status Geographic region/hospital characteristics Comorbidity burden (CHF, CKD, COPD, PVD, AFib, chronic ischemic heart disease) Cardiology consultation (index and post-discharge) LVEF, family history of vascular disease (Fournier only, multivariable, *p*-value only)	Ischemic symptoms (chest pain vs. anginal equivalents vs. silent presentation) Dynamic ECG changes (ST depression/elevation, T-wave inversion) Troponin magnitude and kinetics Known or prior coronary anatomy Hemodynamic instability (shock, pressor requirement) Active bleeding or anemia severity Severity of the precipitating acute illness (e.g., sepsis or respiratory failure severity scores) Renal function as a continuous/graded measure (vs. binary CKD diagnosis) Frailty and life expectancy Echocardiographic regional wall-motion abnormalities Physician-perceived bleeding or procedural risk

## Data Availability

No new data were created in this study. The extraction dataset generated during this review is available from the corresponding author upon reasonable request.

## Supplementary Materials

The following supporting information can be downloaded at: https://www.mdpi.com/article/10.3390/jcm15197586/s1, Supplementary Table S1: Studies excluded at full-text review and study-specific reasons. Supplementary Table S2: Complete database-specific search strategies. Supplementary Table S3: PRISMA 2020 checklist. Supplementary Table S4: QUIPS domain-level decision rules and consensus process. Supplementary Table S5: Full QUIPS risk-of-bias rationale by study and domain.
