# Supplementary material for: Factors Associated with Invasive Coronary Angiography Use in Type 2 Myocardial Infarction: A Systematic Review with Narrative Synthesis
Source: J Clin Med. 2026 Sep 30;15(19):7586. doi: 10.3390/jcm15197586 (PMC13666332; doi:10.3390/jcm15197586)
Supplement: Supplementary file 1 [file jcm-15-07586-s001.zip › jcm-4574977-supplementary.pdf]

Supplementary Materials

Factors Associated With Invasive Coronary Angiography Use in Type 2 Myocardial Infarction: A Systematic Review with Narrative Synthesis

Table S1. Studies Excluded at Full-Text Review, with Reasons

Fifteen studies were excluded at full-text review: ten identified through the database search after meeting title/abstract screening criteria, and five (Tripathi et al., 2021; Smilowitz et al., 2016; Coscia et al., 2022; Chapman et al., 2018; Eggers et al., 2023 [SWEDEHEART]) identified only through citation searching of included and related studies, assessed for eligibility directly without a title/abstract screening stage (Section 3.1; Figure 1 of the main text). Reasons are listed individually below; see Section 3.1 of the main text for the five summary categories these fall into.

Study	Reason for Exclusion
Tripathi et al., 2021	Reported an event-risk/prognostic outcome rather than angiography utilization within T2MI.
McCarthy et al., 2023 (DEFINE-MI)	Coronary CT-angiography/CT-FFR was a protocol-mandated procedure for all participants, not a clinician-selected outcome.
Taggart et al., 2025 (TARGET-Type 2 pilot RCT)	Invasive coronary and structural investigation was assigned by randomization as a trial intervention, not by clinician selection.
Wereski et al., 2022	Compared T1MI to T2MI as groups (risk factors for MI type); did not address within-T2MI factors associated with angiography.
Bularga et al., 2022 (DEMAND-MI)	Coronary and cardiac imaging was protocol-mandated for all participants as part of the study design.
White et al., 2022 (BMJ Open)	An existing meta-analysis that, per its own stated limitations, could not perform multivariable analysis of associated factors due to lack of individual patient data across included studies.
Smilowitz et al., 2016	Compared T2MI to myonecrosis-without-MI as groups; did not address within-T2MI factors associated with angiography.
Sheba Medical Center cohort study	T1MI vs. T2MI group comparison; did not report within-T2MI angiography factors.
McCarthy et al., 2021 (JACC)	T1MI vs. T2MI group comparison of patient characteristics and outcomes; did not report within-T2MI angiography factors.
Coscia et al., 2022 (JAMA Cardiol)	Event-risk/prognostic outcome study; did not report within-T2MI angiography factors.
Raphael et al., 2020 (Circulation)	Community-cohort incidence and trends study reporting event-risk outcomes, not within-T2MI angiography factors.
Chapman et al., 2018 (Circulation)	Long-term outcomes study comparing T1MI/T2MI/myocardial injury groups; did not report within-T2MI angiography factors.
Stein et al., 2014 (PLoS One)	T1MI vs. T2MI characteristics comparison; did not report within-T2MI angiography factors.
Baron et al., 2015 (Heart; TOTAL-AMI)	T1MI vs. T2MI group comparison of long-term mortality; did not report within-T2MI angiography factors.
Eggers et al., 2023 (Sci Rep; SWEDEHEART)	Registry-based trends study comparing T1MI vs. T2MI management over time; did not report within-T2MI angiography factors.

Table S2. Complete Database-Specific Search Strategies

Database	Search Strategy	Date Run	Records Retrieved
PubMed	(("myocardial infarction" AND ("type 2" OR "type II" OR "T2MI")) OR "T2MI" OR "type 2 myocardial infarction" OR "type II myocardial infarction" OR "myocardial oxygen supply-demand mismatch" OR "supply-demand mismatch myocardial infarction") AND ("coronary angiography" OR "invasive management" OR "invasive coronary angiography" OR "cardiac catheterization" OR "revascularization" OR "percutaneous coronary intervention" OR "PCI" OR "conservative management" OR "LHC")	July 16, 2026	1,348
Embase (embase.com)	(("myocardial infarction" AND ("type 2" OR "type II" OR "T2MI")) OR "T2MI" OR "type 2 myocardial infarction" OR "type II myocardial infarction" OR "myocardial oxygen supply-demand mismatch" OR "supply-demand mismatch myocardial infarction") AND ("coronary angiography" OR "invasive management" OR "invasive coronary angiography" OR "cardiac catheterization" OR "revascularization" OR "percutaneous coronary intervention" OR "PCI" OR "conservative management" OR "LHC")	July 16, 2026	4,219
ClinicalTrials.gov	Condition/disease: "type 2 myocardial infarction"; Other terms: T2MI OR "type II myocardial infarction"; Intervention/treatment: left unrestricted for the primary search	July 16, 2026	14

The following search strategies were executed for each database, all restricted to English-language publications from January 1, 2012 to July 16, 2026, using free-text keywords only (no MeSH or Emtree controlled vocabulary terms; adding the sole available heading for myocardial infarction in each thesaurus — MeSH “Myocardial Infarction,” Emtree “heart infarction” — was

tested and increased the PubMed and Embase result counts to 41,587 and 112,175 records respectively without adding type-2-specific discrimination, so neither was retained; see main text Section 4.3). The Embase search was executed via embase.com. Both the PubMed and Embase searches were run on July 16, 2026.

Table S3. PRISMA 2020 Checklist

This checklist maps each PRISMA 2020 reporting item to its location in the main manuscript. Items marked "Not applicable" reflect that no quantitative synthesis was performed in this review (Section 2.7); a certainty-of-evidence assessment was performed despite the absence of pooling, using a modified GRADE approach (Section 3.5; Table 4 of the main text).

Section/Topic	Item #	Checklist Item	Location in Manuscript
Title	1	Identify the report as a systematic review.	Title page
Abstract	2	See the PRISMA 2020 for Abstracts checklist.	Abstract (structured; not separately checked against PRISMA-A)
Rationale	3	Describe the rationale for the review in the context of existing knowledge.	Section 1, paragraphs 1–3
Objectives	4	Provide an explicit statement of the objective(s) or question(s) the review addresses.	Section 1, final paragraph
Eligibility criteria	5	Specify inclusion/exclusion criteria and how studies were grouped for syntheses.	Section 2.2
Information sources	6	Specify all databases, registers, etc. searched, and date each was last searched.	Section 2.3 (databases, date range, and exact search date [July 16, 2026 for PubMed and Embase] specified); Section 2.3 also notes that citation searching of included and related studies was used as a supplementary, non-exhaustive information source (Section 3.1; Figure 1)
Search strategy	7	Present full search strategies for all databases, including filters/limits.	Section 2.3 (verbatim string, language filter, date range, and Embase platform [embase.com] specified). The database search strategy used free-text terms only; see Section 4.3 for the sensitivity limitation this created, evidenced by five relevant studies identified only through citation searching (Section 3.1)
Selection process	8	Specify methods to decide study eligibility, number of reviewers, independence, automation tools.	Section 2.4 (two independent reviewers, third-reviewer adjudication; no automation tool used for eligibility decisions; no formal inter-reviewer agreement statistic calculated)
Data collection process	9	Specify methods to collect data, number of reviewers, independence, automation tools.	Section 2.5 (two independent reviewers; no automation tool used)

Section/Topic	Item #	Checklist Item	Location in Manuscript
Data items – Outcomes	10a	List and define all outcomes sought.	Section 2.2; Table 1 (angiography outcome definition per study)
Data items – Other variables	10b	List and define all other variables sought; describe assumptions about missing data.	Section 2.5 (factor-level extraction template; missing-data handling statement)
Risk of bias assessment	11	Specify methods to assess risk of bias, tool(s) used, reviewers, independence.	Section 2.6 (QUIPS, two independent reviewers; no formal inter-reviewer agreement statistic calculated — see Section 4.3)
Effect measures	12	Specify effect measure(s) used for each outcome.	Section 2.7 (odds ratio, as reported by original studies)
Synthesis methods – eligibility for synthesis	13a	Describe processes to decide which studies were eligible for each synthesis.	Section 2.7
Synthesis methods – data preparation	13b	Describe methods to prepare data for synthesis (missing statistics, conversions).	Section 2.5 (missing-data statement); no data conversion was performed
Synthesis methods – tabulation/display	13c	Describe methods to tabulate or display results.	Tables 1 and 3
Synthesis methods – synthesis approach	13d	Describe synthesis methods and rationale; if meta-analysis, describe model(s) and software.	Section 2.7 (narrative synthesis; rationale for not pooling any factor, including age)
Synthesis methods – heterogeneity exploration	13e	Describe methods to explore heterogeneity (subgroup, meta-regression).	Not applicable — no quantitative synthesis was performed (Section 2.7)
Synthesis methods – sensitivity analyses	13f	Describe sensitivity analyses.	Not applicable — no quantitative synthesis was performed (Section 2.7)
Reporting bias assessment	14	Describe methods to assess risk of bias due to missing results.	Not applicable — no quantitative synthesis was performed (Section 2.7); noted as a limitation (Section 4.3)
Certainty assessment	15	Describe methods to assess certainty in the body of evidence.	Section 2.7 (modified GRADE approach for narrative synthesis: risk of bias, inconsistency, indirectness, and imprecision assessed per factor domain; observational evidence starts at Low certainty per standard GRADE practice)
Study selection	16a	Describe results of search/selection, ideally with a flow diagram.	Section 3.1; Figure 1 (PRISMA 2020 flow diagram, presented with two identification arms: database searching [n = 5,581 identified, 13 assessed for eligibility] and citation searching [n = 5 identified and assessed for eligibility])

Section/Topic	Item #	Checklist Item	Location in Manuscript
Excluded studies	16b	Cite studies that might appear eligible but were excluded, and why.	Section 3.1; Supplementary Table S1, which distinguishes the 10 exclusions identified through the database search from the 5 identified only through citation searching
Study characteristics	17	Cite each included study and present its characteristics.	Table 1
Risk of bias in studies	18	Present risk-of-bias assessments for each included study.	Table 2 (QUIPS)
Results of individual studies	19	Present summary statistics and effect estimates with precision for each study.	Table 3
Results of syntheses – summary	20a	Summarize characteristics/risk of bias among studies contributing to each synthesis.	Not applicable — no quantitative synthesis was performed
Results of syntheses – statistical results	20b	Present results of all statistical syntheses, including heterogeneity if meta-analysis.	Not applicable — no quantitative synthesis was performed
Results of syntheses – heterogeneity investigations	20c	Present results of investigations of heterogeneity.	Not applicable
Results of syntheses – sensitivity analyses	20d	Present results of sensitivity analyses.	Not applicable
Reporting biases	21	Present assessments of risk of bias due to missing results for each synthesis.	Not applicable
Certainty of evidence	22	Present certainty assessments for each outcome.	Section 3.5; Table 4 (certainty rated for all nine factor domains; every factor rated Very Low, with per-factor rationale given in the text following Table 4)
Discussion – interpretation	23a	Provide general interpretation of results in context of other evidence.	Section 4, 4.1
Discussion – limitations of evidence	23b	Discuss limitations of the included evidence.	Section 4.3
Discussion – limitations of review process	23c	Discuss limitations of the review process itself.	Section 4.3 (search date range, English-language restriction, single-reviewer-adjudicated source studies, absence of a formal inter-reviewer agreement statistic, retrospective protocol deposit)
Discussion – implications	23d	Discuss implications for practice, policy, and future research.	Section 4.2
Registration	24a	Provide registration information or state the review was not registered.	Section 2.1 (protocol retrospectively deposited on OSF, DOI 10.17605/OSF.IO/RA3D7, not prospectively registered)
Protocol	24b	Indicate where the protocol can be accessed, or state none was prepared.	Section 2.1; protocol deposited on OSF (retrospective, not prospectively registered)

Section/Topic	Item #	Checklist Item	Location in Manuscript
Amendments	24c	Describe and explain any amendments to the registered protocol.	None; the protocol was deposited retrospectively, after screening and extraction were already complete, as disclosed in Section 2.1
Support	25	Describe sources of financial/non-financial support and the funders' role.	Funding statement (back matter)
Competing interests	26	Declare any competing interests of review authors.	Conflicts of Interest statement (back matter)
Availability of data, code, materials	27	Report which of: data collection forms, extracted data, analytic data, code, other materials are publicly available and where.	Data Availability Statement (back matter); extraction dataset available from corresponding author on reasonable request

Table S4. QUIPS Domain-Level Decision Rules and Consensus Process

Both reviewers rated each QUIPS domain independently using the decision rules below, adapted from standard QUIPS guidance to this review's outcome (angiography utilization rather than prognosis). Independent ratings were compared; concordant ratings were retained, and discordant ratings were resolved by consensus discussion between the two reviewers. No domain rating required adjudication by the third reviewer beyond this consensus step. The applied rules, and the rationale recorded for each study's final rating, are given below; the resulting ratings themselves are presented in Table 2 of the main text.

QUIPS Domain	Rated Low Risk of Bias When	Rated Moderate Risk of Bias When	Rated High Risk of Bias When
Study participation	The study population represents a plausible, minimally restricted sampling frame for the target T2MI population, with no exclusion criterion plausibly related to angiography likelihood.	Some exclusions could plausibly relate to angiography likelihood but affect a small share of the eligible cohort, or their direction of bias is unclear.	Exclusion criteria plausibly created differential angiography-related selection (e.g., excluding patients who died in-hospital or lacked continuous enrollment, since these exclusions correlate with illness severity and procedural eligibility).
Study attrition	Attrition from the analytic sample was zero or minimal (<5%) and unrelated to angiography status or exposure factors, with reasons documented.	Attrition was moderate (5-10%) or its relationship to the outcome/factors was not fully documented.	Attrition was substantial (>10%) or plausibly related to angiography status, without adequate documentation of missing-data handling.
Prognostic factor measurement (here: utilization-associated factor measurement)	Factors were ascertained using validated diagnosis/procedure codes or a documented, consistent case-definition process applied uniformly across the cohort.	Factors were derived from administrative coding or clinician judgment with plausible but unquantified misclassification risk.	Factor ascertainment relied on definitions or adjudication processes with a clear, material risk of misclassification (e.g., a single, non-blinded reviewer classifying an exposure central to the study question).
Outcome measurement	Angiography was ascertained via validated procedure codes (e.g., CPT/ICD-10-PCS/HCPCS) applied consistently across the full cohort, within a clearly bounded assessment window.	Angiography ascertainment involved some coding ambiguity, or the assessment window combined distinct clinical contexts (e.g., index admission and postdischarge periods) without the ability to separate them.	Angiography ascertainment had substantial, undocumented risk of misclassification, or the outcome definition materially conflated distinct clinical events without acknowledgment.
Study confounding	The adjustment set included the pre-specified core confounders for this review (age, sex, comorbidity burden, and an	The adjustment set included some but not all core confounders, without full justification for omissions.	The reported estimate was unadjusted, or the adjustment set omitted multiple core confounders with plausible

	illness-severity surrogate) or justified their omission.		confounding effects on angiography selection.
Statistical analysis and reporting	Effect estimates were reported with point estimates and confidence intervals, model specification was described, and there was no evident selective reporting.	Some model detail or precision (e.g., confidence intervals for a subset of estimates) was missing, or a reported estimate showed an internal inconsistency requiring cautious interpretation.	Effect estimates lacked precision throughout, model specification was not described, or reporting appeared selective.

Table S5. Full QUIPS Risk-of-Bias Rationale by Study and Domain

Table 2 of the main text reports each domain-level rating alone. The complete rationale recorded by the reviewers for each rating, including the specific study features that drove each judgment, is given in full below.

QUIPS Domain	Study	Rating and Full Rationale
Study participation	Smilowitz et al. [2]	Low — 20% stratified national sample of all US hospitals, weighted for national estimates, clear ICD-10-based inclusion.
Study participation	Fournier et al. [3]	High — cohort restricted to patients with no pre-existing heart disease, heart failure, or atrial fibrillation at only 2 French centers; excludes the majority of a typical T2MI population.
Study participation	Goel et al. [7]	Moderate — large national commercial/Medicare Advantage claims cohort with clear inclusion criteria; not generalizable to Medicaid-only or uninsured populations.
Study attrition	Smilowitz et al. [2]	Low — cross-sectional administrative data; no longitudinal follow-up attrition applicable.
Study attrition	Fournier et al. [3]	Low — 104/6,480 (1.6%) excluded for missing data, with documented reasons; no longitudinal attrition.
Study attrition	Goel et al. [7]	Moderate — patients who died in-hospital, were discharged to hospice, or lacked 6 months of continuous enrollment were excluded from the procedure/consultation models, which may selectively remove the sickest patients least likely to undergo angiography.
Prognostic factor measurement	Smilowitz et al. [2]	Moderate — factors (insurance, region, hospital type) are objective administrative variables; comorbidities rely on ICD-10 coding accuracy.
Prognostic factor measurement	Fournier et al. [3]	High — type 2 ACS diagnosis and factor extraction were adjudicated by a single, unblinded reviewer from retrospective chart review, with no reported inter-rater reliability.
Prognostic factor measurement	Goel et al. [7]	Moderate — factors derived from claims/ICD-10 coding; subject to the same T2MI coding-validity uncertainty the authors themselves acknowledge as a limitation.
Outcome measurement	Smilowitz et al. [2]	Low — angiography ascertained via standardized ICD-10/CCSR procedure codes.
Outcome measurement	Fournier et al. [3]	Moderate — exploration status and invasive/non-invasive classification extracted from medical records; objective but manually abstracted.

Outcome measurement	Goel et al. [7]	Low — angiography ascertained via CPT/ICD-10-PCS/HCPCS codes; standard claims-based approach with general claims-completeness limitations.
Study confounding	Smilowitz et al. [2]	Moderate — extensive covariate adjustment (demographics, comorbidities, hospital and geographic factors), but administrative data lacks clinical granularity (no labs, imaging, symptom severity).
Study confounding	Fournier et al. [3]	High — multivariable model restricted to variables with univariate $p<0.20$, and authors explicitly state a fuller model would have been unstable given the small event count (n=78 explored of 224).
Study confounding	Goel et al. [7]	Moderate — most extensive adjustment set of the three studies (~20 covariates), though still limited by the same lack of clinical granularity inherent to claims data.
Statistical analysis and reporting	Smilowitz et al. [2]	Low — clear methodology, multivariable logistic regression with ORs/CIs fully reported, large sample well powered.
Statistical analysis and reporting	Fournier et al. [3]	Moderate — the multivariable result for age is directionally discordant with the study's own univariate finding; the published report does not permit resolution of this discrepancy, so the estimate was excluded from synthesis rather than interpreted.
Statistical analysis and reporting	Goel et al. [7]	Low — clear, detailed reporting across multiple forest-plot/heat-map figures with adjusted ORs and CIs for every factor and outcome examined.
